# Two new mites of *Armascirus* Den Heyer, 1978 (Trombidiformes, Cunaxidae) from Chinese fauna

**DOI:** 10.3897/zookeys.1267.172611

**Published:** 2026-01-27

**Authors:** Jian-Xin Chen, Mao-Yuan Yao, Tian-Lei Liu, You-Fang Wu, Jian-Jun Guo, Dao-Chao Jin

**Affiliations:** 1 College of Agriculture, Anshun University, Anshun, 561000, China Anshun University Anshun China; 2 Institute of Entomology, Guizhou University, Guiyang, Guizhou, 550025, China Guizhou University Guiyang China; 3 Guizhou Provincial Key Laboratory for Agricultural Pest Management of the Mountainous Region, Guiyang, Guizhou, 550025, China Scientific Observing and Experimental Station of Crop Pest in Guiyang, Ministry of Agriculture and Rural Affairs of the P.R. China Guiyang China; 4 Scientific Observing and Experimental Station of Crop Pest in Guiyang, Ministry of Agriculture and Rural Affairs of the P.R. China, Guiyang 550025, China Guizhou Provincial Key Laboratory for Agricultural Pest Management of the Mountainous Region Guiyang China

**Keywords:** Cunaxinae, morphology, predatory mites, taxonomy

## Abstract

In this study, two new species, *Armascirus kuandianensis* Chen & Jin, **sp. nov**. and *A. stellatus* Chen & Jin, **sp. nov**. are described and illustrated based on females. Additionally, a key to adult females of *Armascirus* species of China is provided.

## Introduction

Predatory mites in the family Cunaxidae are found worldwide, except in Antarctica (Smiley 1992; Skvarla et al. 2014). These mites serve as important ecological regulators in terrestrial ecosystems by preying on nematodes, fungal spores, fungus gnats, springtails, and other small soil-inhabiting organisms (Skvarla et al. 2014; Hernandes et al. 2015).

Currently, the family Cunaxidae includes 32 genera and over 450 described species globally (Wurlitzer et al. 2022; Khaustov and Khaustov 2024; Mirza et al. 2025; Chen et al. 2025). The genus *Armascirus* Den Heyer, 1978 is particularly diverse, with 51 species recorded worldwide. However, in China, only five species have been recorded (*A. taurus* (Kramer, 1881), *A. bison* (Berlese, 1888), *A. jini* Liu, Yi & Guo, 2015, *A. apophysis* Chen & Jin, 2021, and *A. yulongensis* Chen & Jin, 2021). These species are distributed across Guizhou, Sichuan, Yunnan, Xizang (Tibet), Shanghai, Fujian, Jiangsu, Beijing, and Taiwan (Lin and Zhang 2010; Chen et al. 2021). This limited documentation suggests that many *Armascirus* species in China remain to be found or are undescribed, highlighting the need for further taxonomic research.

In this study, we report two new species of *Armascirus* from China, namely, *A. kuandianensis* Chen & Jin, sp. nov. and *A. stellatus* Chen & Jin, sp. nov. These discoveries bring number of *Armascirus* species in China to seven, significantly improving our understanding of cunaxid mite diversity in the Chinese fauna.

## Materials and methods

Mite samples were isolated using modified Berlese-Tullgren funnels for 8–12 hours, preserved in 75% alcohol, and then mounted in Hoyer’s medium on slides (Walter and Krantz 2009). Geographic coordinates and altitudes were obtained by a smartphone with GPS. Line drawings were prepared with the aid of a drawing tube attached to a phase contrast and DIC Nikon Ni E microscope. Photographs were taken using a camera (Nikon DS-Ri 2) attached to the Nikon Ni E microscope and figures were edited with Adobe Photoshop CC 2019. The length of the gnathosoma was measured from the base to the top of the subcapitulum, the length of the idiosoma, from the suture between the gnathosoma and idiosoma to the posterior margin of the idiosoma, the width of the idiosoma at its broadest level and the length of the legs from the ventral insertion of coxae to the tip of the claw. All measurements were taken in micrometers (µm) using Nikon NIS Elements AR 4.50 software and provided for the holotype and the paratypes. The dorsal and ventral setal nomenclature follows Fisher et al. (2011), Skvarla et al. (2014), and Paktinat-Saeij et al. (2017); setal notation on legs follows Den Heyer (1981). The species were identified or compared using the keys of Kalúz (2009) and Skvarla et al. (2014). All types are deposited in the Institute of Entomology, Guizhou University, Guiyang, P.R. China (GUGC).

Abbreviations: prodorsum: anterior trichobothria (*at*), posterior trichobothria (*pt*), lateral proterosomal (*lps*), median proterosomal (*mps*); hysterosoma: internal humerals (*c_1_*), external humerals (*c_2_*), internal dorsals (*d_1_*), internal lumbals (*e_1_*), internal sacrals (*f_1_*), internal clunals (*h_1_*), external clunals (*h_2_*); venter: propodogastral seta (*ppgs*), hysterogastral seta (*hgs*); anal region: pseudanal (*ps*); genital region: genitals (*g*); gnathosoma: hypognathals (*hg*), adoral setae (*ads*); leg: attenuate (sharply) solenidion (*asl*), blunt-pointed rod-like solenidion (*bsl*), famulus (*fam*), trichobothria (*T*), simple tactile seta (*sts*), microseta (*mst*), dorsoterminal solenidion (*dtsl*).

## Results

### Family Cunaxidae Thor, 1902

**Subfamily Cunaxinae Den Heyer, 1978**

#### Armascirus

Taxon classificationAnimaliaTrombidiformesCunaxidae

Den Heyer, 1978

C68E7B1E-614D-5C1E-A025-BBD7F18E038F

##### Generic diagnosis.

see Skvarla et al. (2014).

##### Type species.

*Armascirus huyssteeni* Den Heyer, 1978

#### Armascirus kuandianensis

Taxon classificationAnimaliaTrombidiformesCunaxidae

Chen & Jin sp. nov.

CE630FC9-E6A8-506D-9D55-6CB553864A6D

https://zoobank.org/173B7537-9907-4A4E-AD58-9D4A1F96453F

Figs 1, 2, 3, 4, 5, 6, 7, 8, 9, 10, 11, 12, 13, 14, 15, 16

##### Diagnosis.

**Female**. Proterosomal and hysterosomal (median) shield present; palp telofemur with one apophysis; subcapitulum with papillae, area lateral to *hg_3_* and *hg_4_* with reticulations; coxae I–IV with reticulations; propodogastral setae (*ppgs*) on small platelets.

##### Description.

**Female** (*n =* 3). Idiosoma 755 (755–760) long, 477 (477–537) wide.

**Figure 1.**
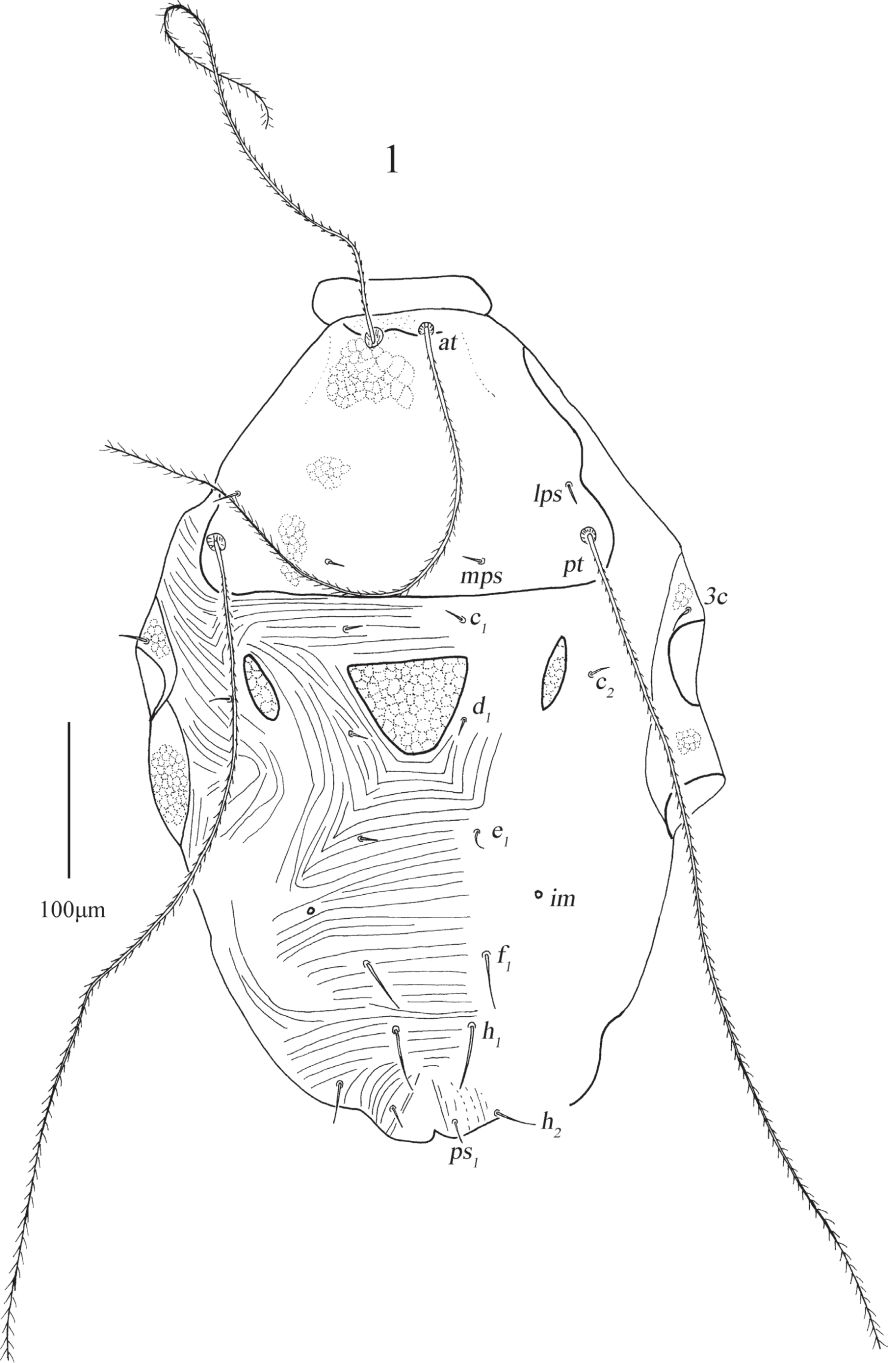
*Armascirus kuandianensis* Chen & Jin, sp. nov., female holotype: dorsal idiosoma.

***Dorsum*** (Figs 1, 3–5). Proterosomal shield 186 (186–190) long, 219 (219–272) wide, and covered with reticulations, bearing two pairs of trichobothria (*at* and *pt*), two pairs of tactile setae (*lps* and *mps*), *lps* closer to *pt*, and *pt* longer than *at*. Hysterosomal (median) shield inverted triangle 63 (63–74) long, 84 (84–98) wide, and covered with reticulations, one pair of small lateral plates 51 (51–58) long, 13 (13–16) wide, and also covered with reticulations; except for median shield and lateral plates, hysterosomal dorsum soft and striated, area between proterosomal shield and median shield transverse striae, lateral area outside median shield with lengthwise striation, area between median shield and *h_1_* with transverse striae; and with seven pairs of simple setae (*c_1_*, *c_2_*, *d_1_*, *e_1_*, *f_1_*, *h_1_*, and *h_2_*) and one pair of lyrifissures (*im*) situated anterolaterally to *f_1_*. Setal lengths and distances: *at* 405 (355–405), *pt* 559 (530–559), *lps* 18 (16–18), *mps* 18 (16–18), *c_1_* 16 (12–16), *c_2_* 14 (14–14), *d_1_* 15 (14–15), *e_1_* 13 (13–16), *f_1_* 46 (43–46), *h_1_* 50 (50–51), 32 (30–39); *at*–*at* 35 (33–35), *pt*–*pt* 239 (239–253), *lps*–*lps* 212 (212–221), *mps*–*mps* 127 (127–133), *lps*–*mps* 73 (73–80), *at*–*lps* 138 (138–138), *pt*–*mps* 72 (72–75), *pt*–*lps* 35 (34–35), *at*–*mps* 156 (150–156), *at*–*pt* 173 (170–173), *c_1_*–*c_1_* 75 (75–96), *c_2_*–*c_2_* 234 (234–253), *d_1_*–*d_1_* 73 (73–108), *e_1_*–*e_1_* 62 (62–109), *f_1_*–*f_1_* 77 (77–103), *h_1_*–*h_1_* 47 (47–55), *c_1_*–*c_2_* 88 (88–144), *c_1_*–*d_1_* 64 (64–87), *c_2_*–*d_1_* 85 (85–150), *d_1_*–*e_1_* 72 (72–93), *e_1_*–*f_1_* 80 (80–111), *f_1_*–*h_1_* 49 (49–58).

**Figure 2.**
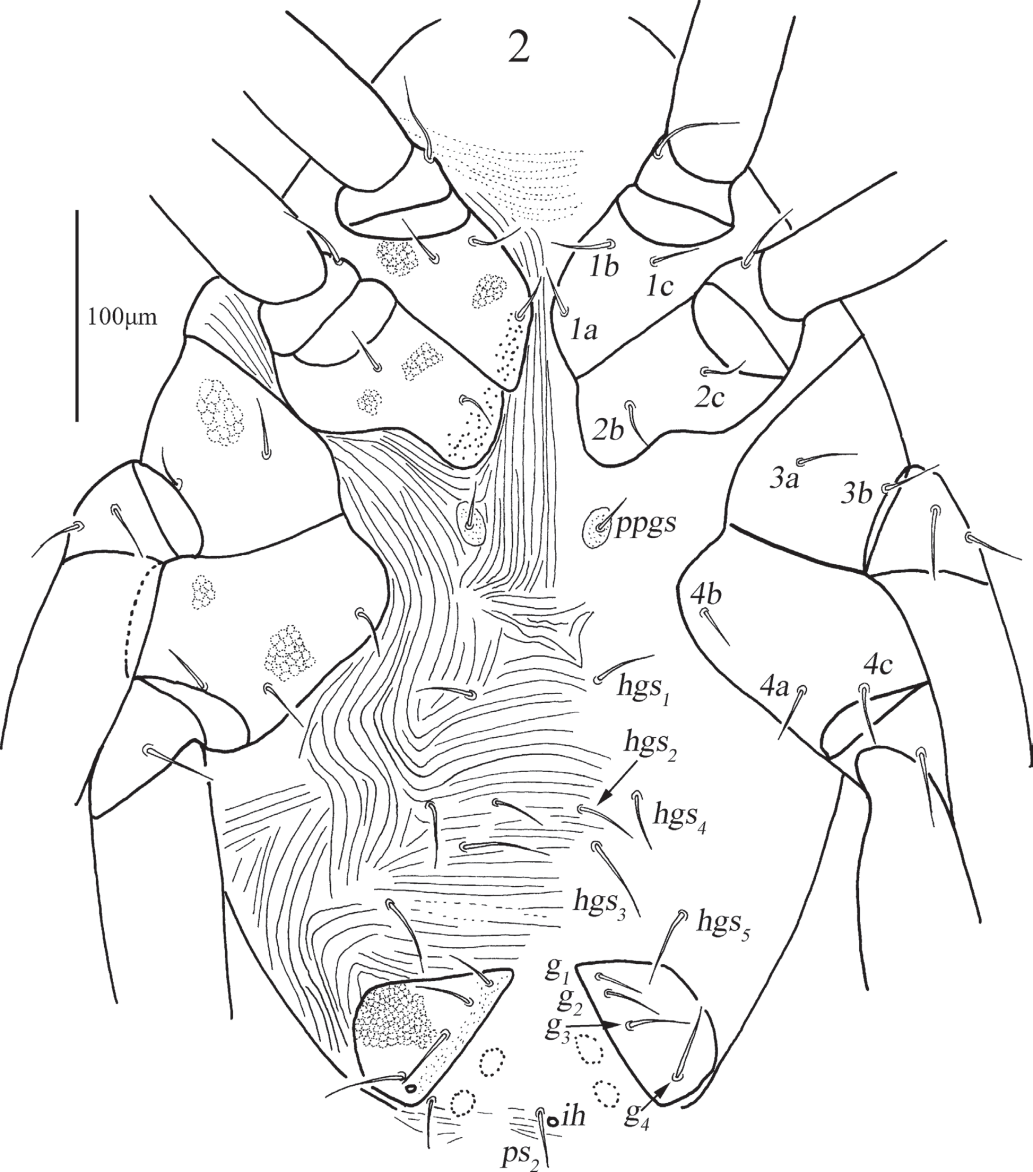
*Armascirus kuandianensis* Chen & Jin, sp. nov., female holotype: ventral idiosoma.

***Venter*** (Figs 2, 6–7). Ventral area between coxae I and gnathosoma with transverse striae. Coxae I–II with reticulations and papillae, whereas coxae III–IV only with reticulations; centrally, between coxae I–IV plates with longitudinal striae anterior to *hgs_1_*; areas between *hgs_1_* and genital plates with transverse striae and outside with lengthwise striation. Setal formula of coxal plates I–IV: 3(*1a*–*c*)-2(*2b*–*c*)-3(*3a*–*c*)-3(*4a*–*c*) *sts*; one pair of propodogastral setae (*ppgs*) 23 (23–25) in length and present on small platelets that with dot-like papillae, five pairs of hysterogastral setae (*hgs_1_–hgs_5_*), 33 (31–33), 35 (33–35), 53 (43–53), 36 (36–50) and 51 (48–51) in length, respectively. Genital plates with papillae and reticulations, two pairs of visible genital papillae and four pairs of genital setae (*g_1_*–*g_4_*) that 32 (32–33), 36 (36–36), 40 (38–40) and 42 (39–42) in length, respectively. Anal region with two pairs of pseudanal setae (*ps_1_–ps_2_*), 21 (14–21) and 32 (28–32) in length, respectively, and one pair of lyrifissures (*ih*) close to *ps_2_*.

**Figures 3–5.**
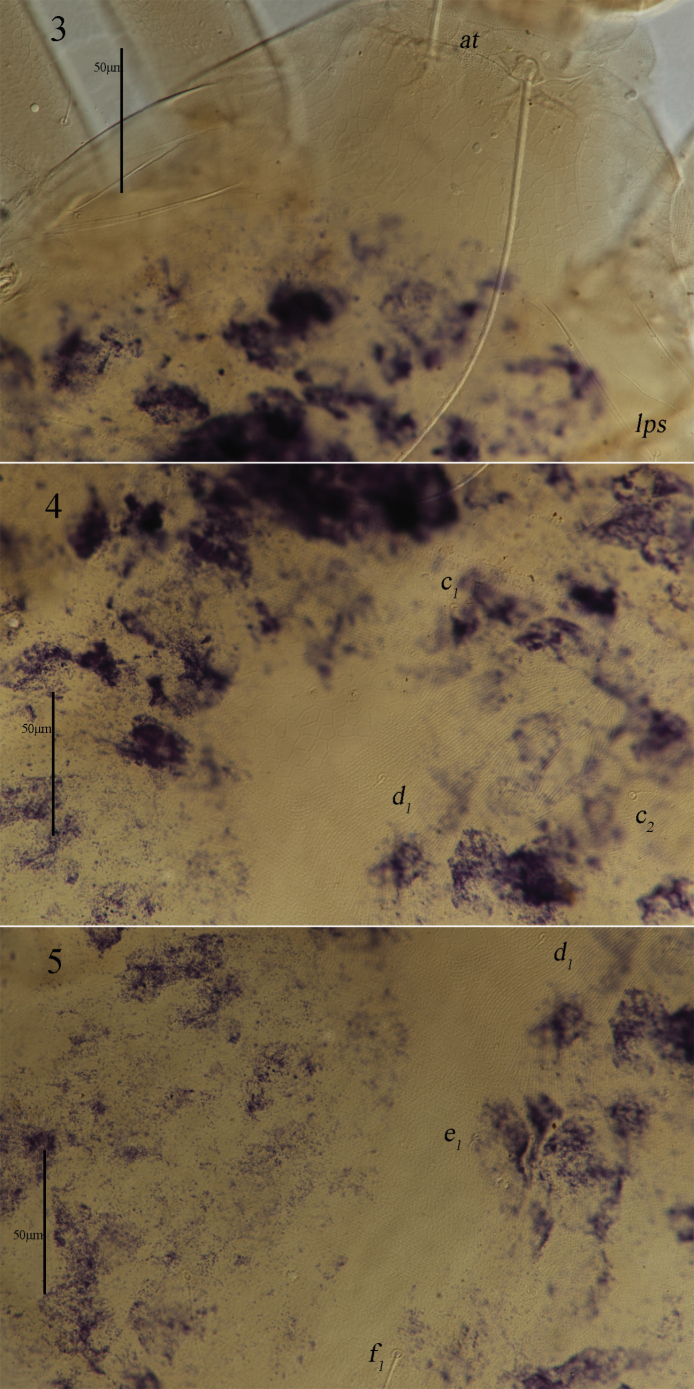
*Armascirus kuandianensis* Chen & Jin, sp. nov., female holotype: dorsal idiosoma.

**Figures 6–9.**
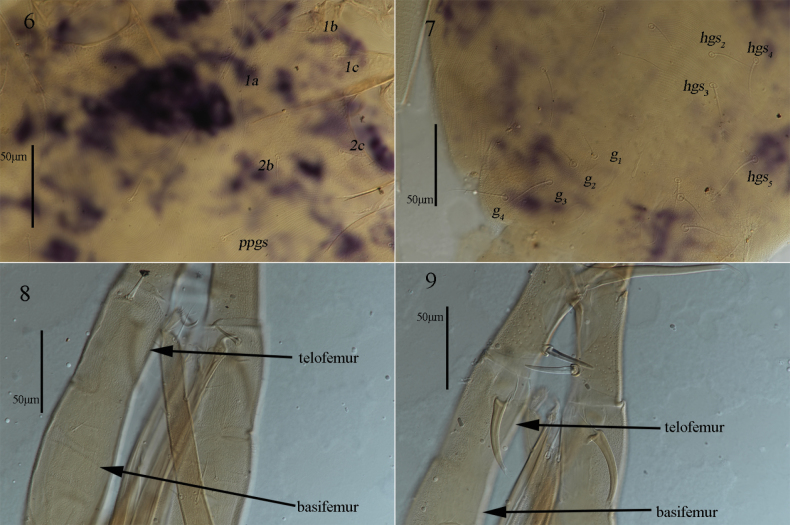
*Armascirus kuandianensis* Chen & Jin, sp. nov., female holotype. **6, 7**. Ventral idiosoma; **8, 9**. Palp.

**Figures 10–12.**
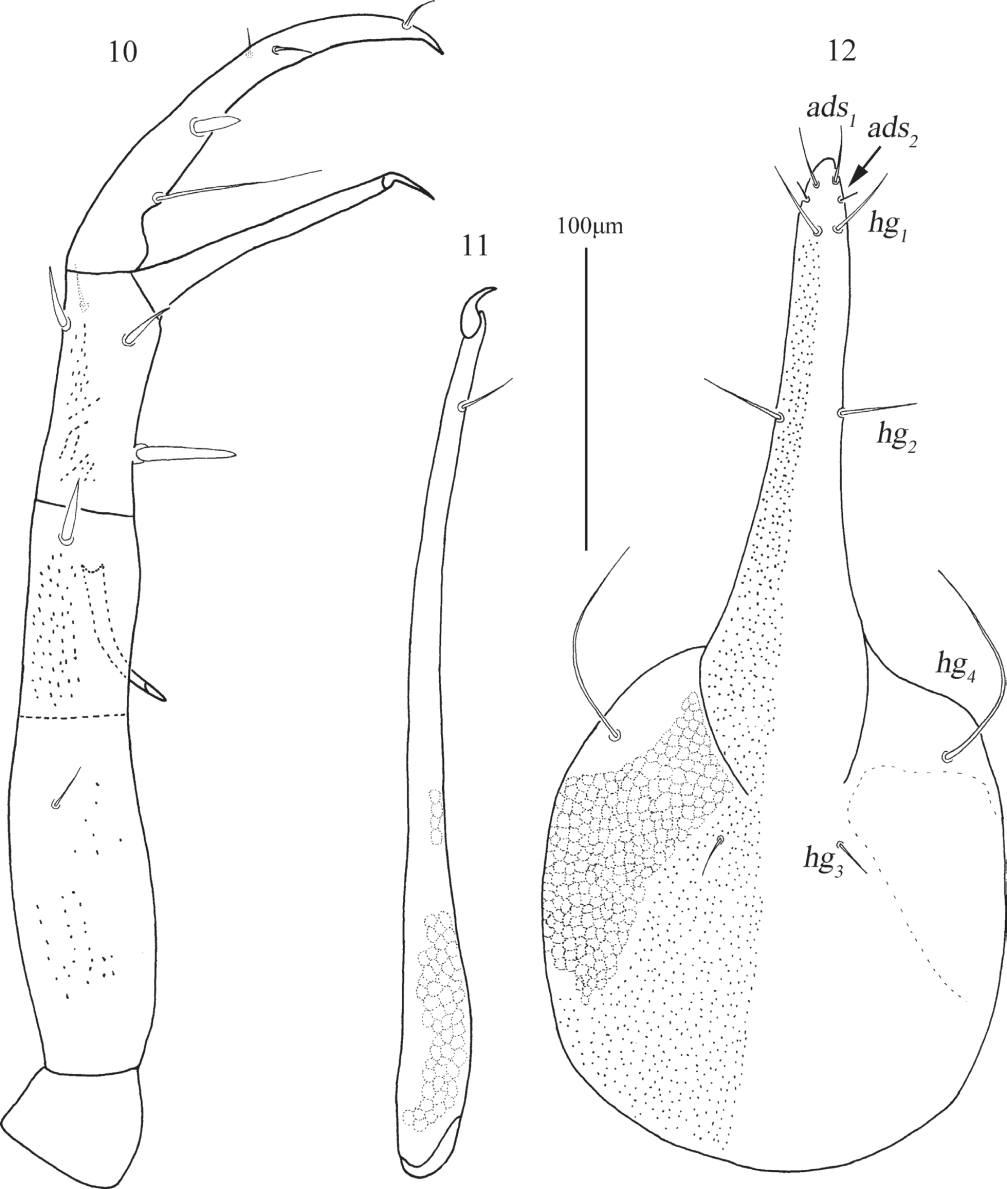
*Armascirus kuandianensis* Chen & Jin, sp. nov., female holotype. **10**. Palp; **11**. Chelicerae; **12**. Subcapitulum.

***Gnathosoma*** (Figs 8, 9, 10). Palp (Figs 8, 9, 10). Five-segmented, 436 (425–436) long, all segments with papillae. Palp chaetotaxy: trochanter none; basifemur with one simple seta; telofemur with one spine-like seta and one apophysis; genu with one simple seta, three spine-like setae (one of which is stout near inner base) and one elongate pointed apophysis; tibiotarsus one spine-like seta, three simple setae (one longer near inner base) and one distal solenidion; claw well developed. Chelicera (Fig. 11). 282 (282–282) long, covered by reticulations; cheliceral seta 21 (21–24) in length; chela developed. Subcapitulum (Fig. 12). 322 (322–324) long, 158 (141–158) wide and with papillae, area lateral to *hg_3_* and *hg_4_* with reticulations; two pairs of short adoral setae, *ads_1_–ads_2_*, 20 (20–23) and 6 (6–9) in length; four pairs of hypostomal setae, *hg_1_–hg_4_*, 31 (31–31), 38 (38–40), 15 (15–18) and 89 (88–89) in length, respectively. Distances of *hg* setae: *hg_1_*–*hg_1_* 8 (7–8), *hg_2_*–*hg_2_* 19 (19–20), *hg_3_*–*hg_3_* 38 (38–39), *hg_4_*–*hg_4_* 109 (105–117), *hg_1_*–*hg_2_* 62 (62–76), *hg_2_*–*hg_3_* 153 (153–168), *hg_3_*–*hg_4_* 51 (48–51).

**Figures 13–16.**
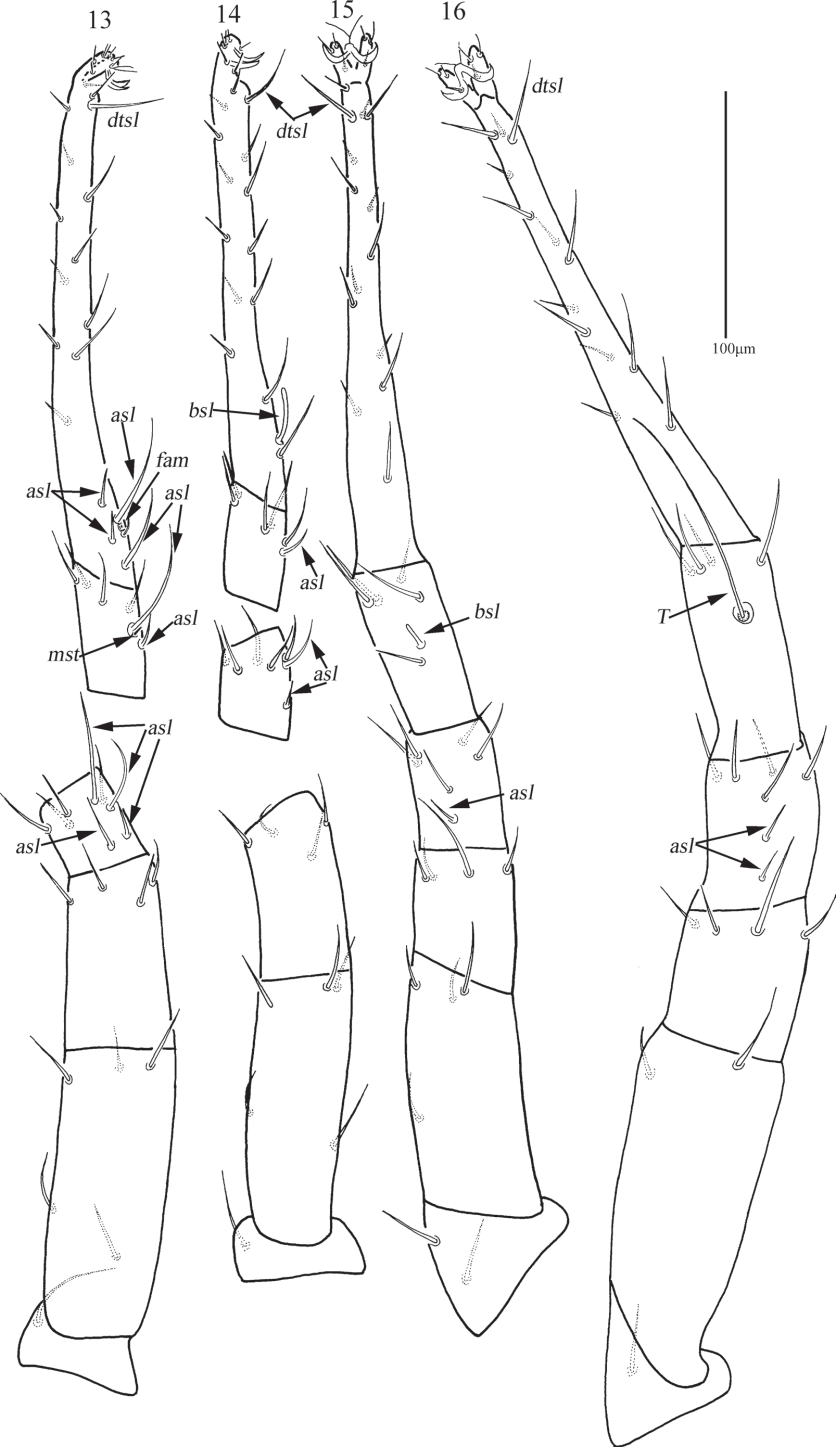
*Armascirus kuandianensis* Chen & Jin, sp. nov., female holotype: leg I–IV.

***Legs*** (Figs 13–16). With reticulations, lengths of leg I–IV: 474 (474–507), 404 (404–466), 509 (509–524), 559 (541–559); lengths of tarsus I–IV: 192 (179–195), 172 (167–180), 207 (194–207), 206 (197–210). *T* on tibia IV 111 (110–112) in length. Legs I–IV chaetotaxy: coxae I–IV 3-2-3-3 *sts*; trochanters I–IV 1-1-2-1 *sts*; basifemora I–IV 5-5-4-2 *sts*; telofemora I–IV 4-4-4-4 *sts*. Genu I 4 *asl*, 4 *sts*; genu II 2 *asl*, 5 *sts*; genu III 1 *asl*, 5 *sts*; genu IV 2 *asl*, 5 *sts*. Tibia I 1 *asl*, {1 *asl*, 1 *mst*}, 4 *sts*; tibia II 1 *asl*, 5 *sts*; tibia III 1 *bsl*, 5 *sts*; tibia IV 1 smooth *T*, 4 *sts*. Tarsus I 4 *asl*, 1 *fam*, 1 *dtsl*, 20 *sts*; tarsus II 1 *bsl*, 1 *dtsl*, 19 *sts*; tarsus III 1 *dtsl*, 19 *sts*; tarsus IV 1 *dtsl*, 18 *sts*.

##### Other developmental stages.

Unknown.

##### Etymology.

The name of this new species is derived from the name of the county in which the type locality is located: Kuandian County.

##### Remarks.

The new species resembles *A. cerris* Kalúz, 2009 (adult female) with having hysterosomal median shield and one pair of small lateral platelets, and palp telofemur only with one apophysis. However, it can be distinguished from *A. cerris* by the following characteristics: (1) two pairs of genital papillae (vs three in *A. cerris*); (2) coxae I–II with reticulations and papillae, coxae III–IV only with reticulations (vs coxae I–II and III–IV contiguous and smooth in *A. cerris*); (3) one pair of propodogastral setae (*ppgs*) present on small platelets (vs integument in *A. cerris*).

##### Material examined.

***Holotype***: China • 1 ♀ (slide no. LN-CU-2010080101) Hekou Village, Changtian Town, Kuandian County, Dandong City, Liaoning Province; 40°29'6"N, 124°48'49" E; 102 m a.s.l.; 1 August. 2010; collected from fallen leaves by Li-Xia Xie, Da-Xing Yang, Rong Huang and Bin Li. ***Paratypes***: China • 2 ♀ (LN-CU-2010080102–LN-CU-2010080103), the same data as for holotype.

##### Distribution.

Known only from the type locality.

#### Armascirus stellatus

Taxon classificationAnimaliaTrombidiformesCunaxidae

Chen & Jin sp. nov.

4742E23F-C945-5F1D-8491-549E1FD95CC7

https://zoobank.org/F18C27A2-982C-49F9-92B1-F8D590CDBF6C

Figs 17, 18, 19, 20, 21, 22, 23, 24, 25, 26, 27, 28, 29, 30, 31, 32

##### Diagnosis.

**Female**. Proterosomal and median shields are covered with star-shaped reticulations, which are composed of papillae and reticulations, resembling stars connecting tracks (Figs 19, 21–23). The median shield large and with three pairs of simple setae (*c_1_*, *d_1_*, and *e_1_*), with no lateral plates present; palp basifemur with one short pointed apophysis, telofemur two elongate apophyses.

##### Description.

**Female** (*n =* 4). Idiosoma long 507 (507–600), wide 374 (374–441).

**Figure 17.**
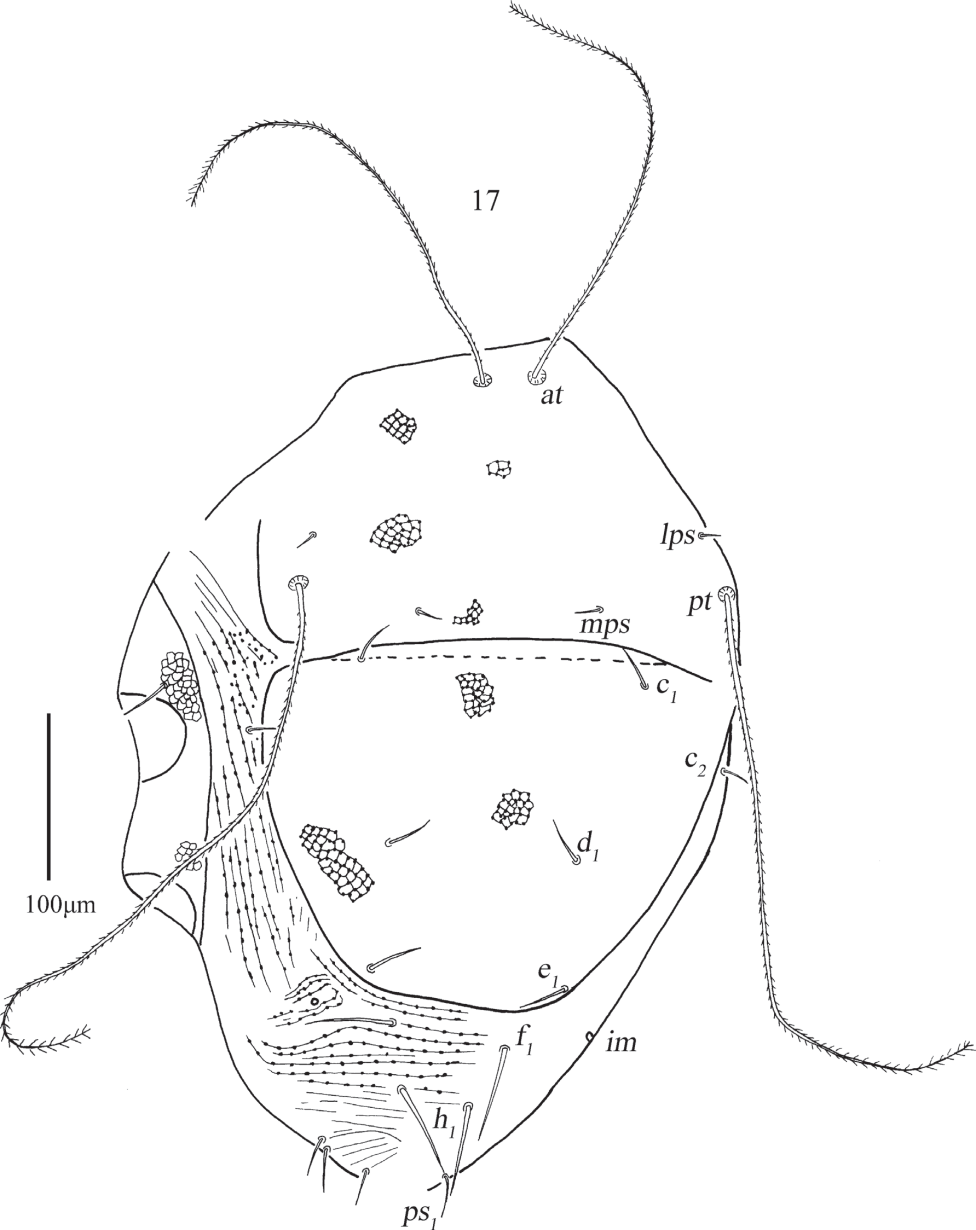
*Armascirus stellatus* Chen & Jin, sp. nov., female holotype: dorsal idiosoma.

***Dorsum*** (Figs 17, 19–21). Proterosomal shield 166 (157–166) long, 275 (213–275) wide, and covered with star-shaped reticulations, which consists of papillae (4–7, with 5 or 6 most common) and reticulation, resembling stars connecting tracks (Figs 19, 21, 22). It bears two pairs of trichobothria (*at* and *pt*), two pairs of tactile setae (*lps* and *mps*), *lps* closer to *pt* than *at*, *pt* longer than *at*. Hysterosomal (median) shield 223 (173–223) long, 266 (213–266) wide, and covered with star-shaped reticulations and with three pairs of simple setae (*c_1_*, *d_1_*, *e_1_*); median shield laterally flanked by longitudinal striae with papillae, area between median shield and *h_1_* with transverse striae with papillae. Setae *f_1_*, *h_1_*, and one pair of lyrifissures (*im*) situated on integument. Setal lengths and distances: *at* 293 (291–300), *pt* 393 (393–452), *lps* 18 (14–18), *mps* 20 (12–20), *c_1_* 32 (28–32), *c_2_* 20 (20–22), *d_1_* 34 (27–34), *e_1_* 43 (37–43), *f_1_* 62 (54–62), *h_1_* 68 (56–68); *at*–*at* 32 (32–37), *pt*–*pt* 256 (245–256), *lps*–*lps* 231 (219–231), *mps*–*mps* 107 (100–107), *lps*–*mps* 76 (76–77), *at*–*lps* 133 (122–133), *pt*–*mps* 68 (71–68), *pt*–*lps* 31 (31–36), *at*–*mps* 139 (133–139), *at*–*pt* 158 (156–158), *c_1_*–*c_1_* 171 (140–171), *c_2_*–*c_2_* 276 (267–276), *d_1_*–*d_1_* 110 (99–110), *e_1_*–*e_1_* 109 (98–109), *f_1_*–*f_1_* 70 (63–70), *h_1_*–*h_1_* 42 (42–44), *c_1_*–*c_2_* 80 (78–80), *c_1_*–*d_1_* 110 (99–110), *c_2_*–*d_1_* 106 (100–106), *d_1_*–*e_1_* 76 (68–76), *e_1_*–*f_1_* 35 (35–52), *f_1_*–*h_1_* 41 (38–41).

**Figure 18.**
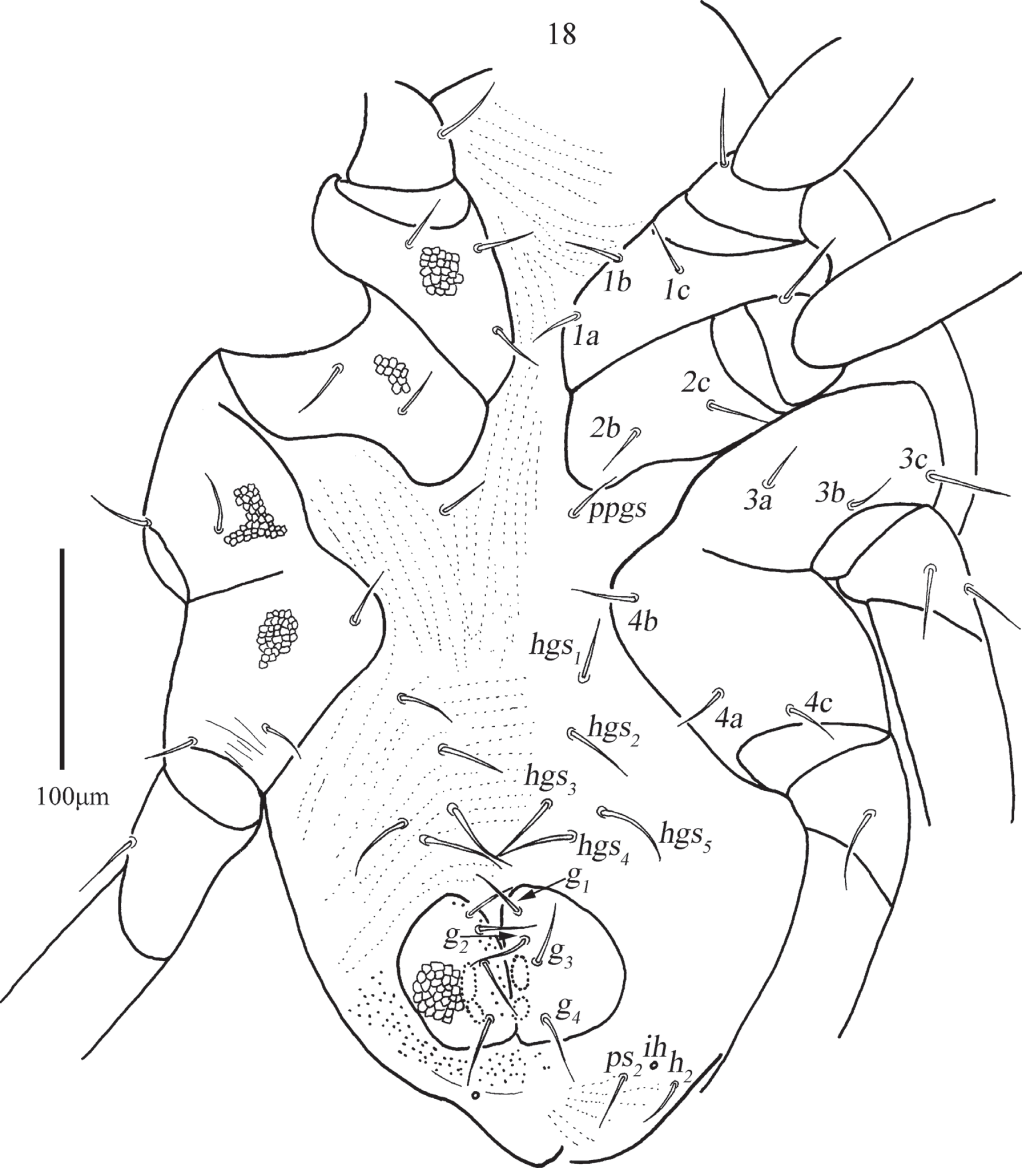
*Armascirus stellatus* Chen & Jin, sp. nov., female holotype: dorsal idiosoma.

**Figures 19–25.**
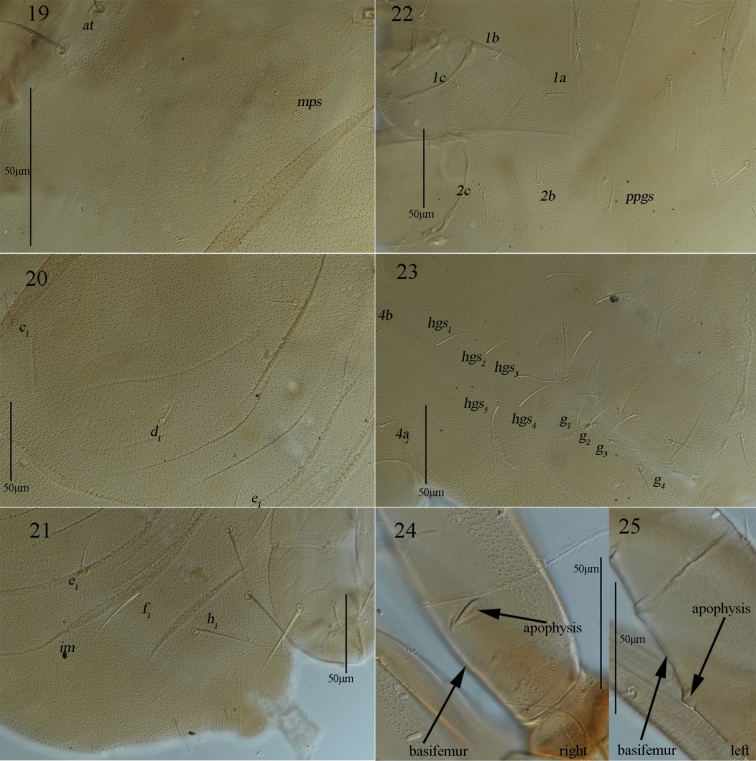
*Armascirus stellatus* Chen & Jin, sp. nov., female holotype. **19–21**. Dorsal idiosoma; **22, 23**. Ventral idiosoma; **24, 25**. Palp.

***Venter*** (Figs 18, 22, 23). Coxae I–IV with reticulations, area between coxae I–II plate groups with longitudinal striae; area between *ppgs* and *hgs_1_* with longitudinal striae; areas between *hgs_1_* and genital plates with transverse striae. Setal formula of coxal plates I–IV: 3(*1a*–*c*)-2(*2a*, *2c*)-3(*3a*–*c*)-3(*4a*–*c*) *sts*; one pair of propodogastral setae (*ppgs*), 28 (28–35) in length, and five pairs of hysterogastral setae (*hgs_1_*–*hgs_5_*), 32 (32–39), 37 (37–42), 41 (41–43), 43 (43–44) and 44 (44–46) in length. Genital plates with reticulations and papillae, two pairs of visible genital papillae and four pairs of genital setae (*g_1_*–*g_4_*) that 30 (27–30), 36 (34–36), 31 (31–32) and 34 (34–36) in length, respectively. Anal region with longitudinal striae, two pairs of pseudanal setae (*ps_1_*–*ps_2_*), 28 (22–28) and 30 (24–30) in length, and one pair of lyrifissures (*ih*).

**Figures 26–28.**
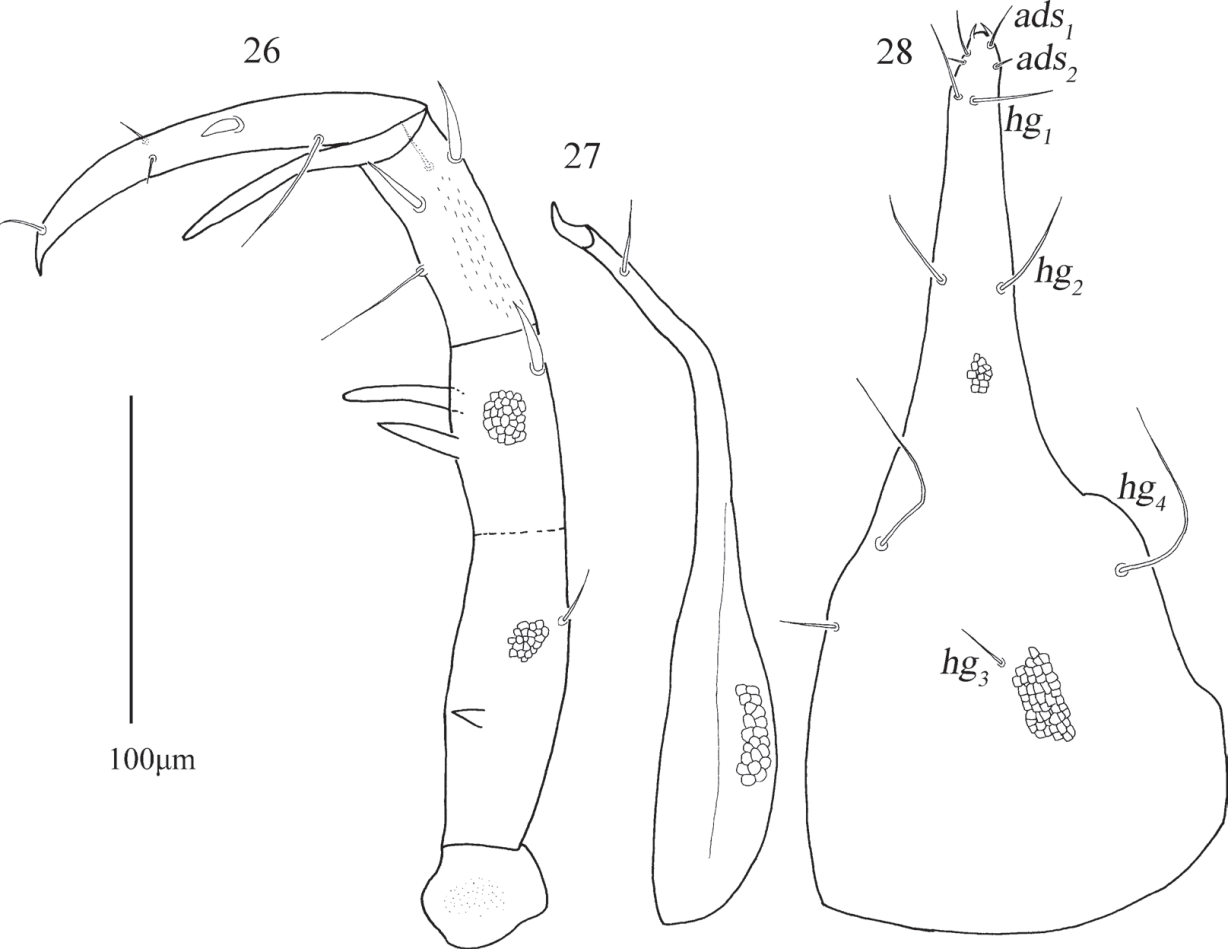
*Armascirus stellatus* Chen & Jin, sp. nov., female holotype. **26**. Palp; **27**. Chelicerae; **28**. Subcapitulum.

***Gnathosoma*** (Figs 24, 25, 26, 27, 28). Palp (Figs 24, 25, 26). Five-segmented, 399 (370–399) long, basifemur and telofemur with reticulations. Palp chaetotaxy: trochanter none; basifemur one dorsal simple seta and one short pointed apophysis; telofemur one dorsal spine-like seta, one pointed apophysis and one blunt apophysis; genu two spine-like setae, two simple setae and one elongate pointed apophysis; tibiotarsus three simple setae, one of which is longer near the inner base, one spine-like seta and one distal solenidion; claw well developed. Chelicera (Fig. 27). 265 (254–265) long, with reticulations and papillae; cheliceral seta 23 (22–25) in length; chela developed. Subcapitulum (Fig. 28) 303 (303–312) long, 143 (143–156) wide, with reticulations; two pairs of short adoral setae, *ads_1_*–*ads_2_*, 20 (20–20) and 8 (5–8) in length; four pairs of hypostome setae, *hg_1_*–*hg_4_*, 31 (30–31), 48 (40–48), 17 (17–20) and 83 (83–84) in length, respectively. Distances of *hg* setae: *hg_1_*–*hg_1_* 6 (6–7), *hg_2_*–*hg_2_* 22 (22–25), *hg_3_*–*hg_3_* 57 (50–57), *hg_4_*–*hg_4_* 83 (83–113), *hg_1_*–*hg_2_* 60 (60–62), *hg_2_*–*hg_3_* 112 (103–112), *hg_3_*–*hg_4_* 50 (43–50).

**Figures 29–32.**
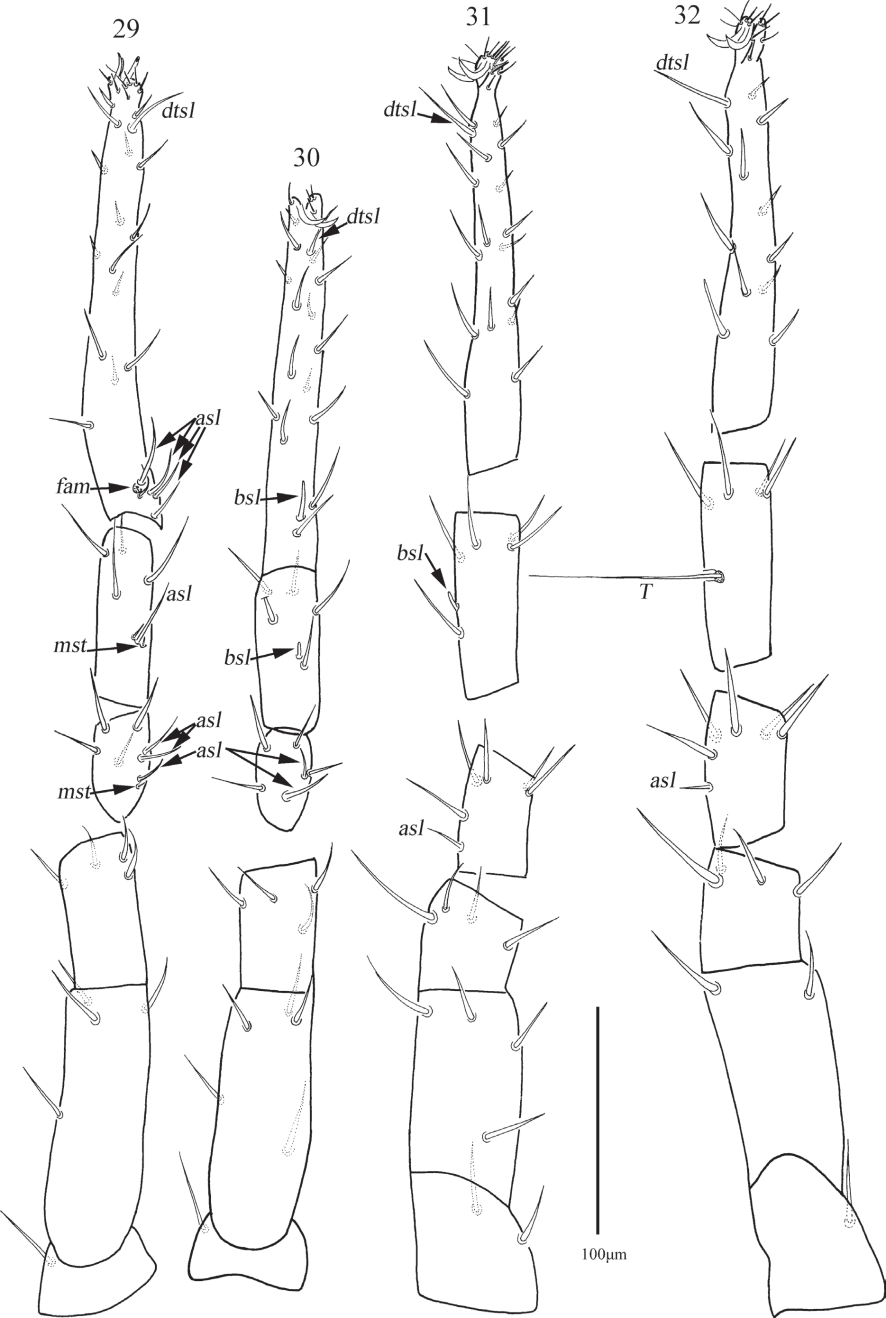
*Armascirus stellatus* Chen & Jin, sp. nov., female holotype: leg I–IV.

***Legs*** (Figs 29–32). With reticulations, lengths of leg I–IV: 515 (503–515), 450 (450–480), 507 (490–507), 521 (521–525); lengths of tarsus I–IV: 187 (184–187), 156 (156–163), 178 (165–178), 173 (173–175). *T* on tibia IV 93 (88–93) in length. Legs I–IV chaetotaxy: Coxae I–IV 3-2-3-3 *sts*; trochanters I–IV 1-1-2-1 *sts*; basifemora I–IV 4-5-4-2 *sts*; telofemora I–IV 4-4-4-4 *sts*. Genu I 2 *asl*, {1 *asl*, 1 *mst*}, 4 *sts*; genu II 2 *asl*, 4 *sts*; genu III 1 *asl*, 5 *sts*; genu IV 1 *asl*, 5 *sts*. Tibia I {1 *asl*, 1 *mst*}, 4 *sts*; tibia II 1 *asl*, 5 *sts*; tibia III 1 *bsl*, 5 *sts*; tibia IV 1 smooth *T*, 4 *sts*. Tarsus I 4 *asl*, 1 *fam*, 1 *dtsl*, 23 *sts*; tarsus II 1 *bsl*, 1 *dtsl*, 19 *sts*; tarsus III 1 *dtsl*, 26 *sts*; tarsus IV 1 *dtsl*, 19 *sts*.

##### Other developmental stages.

Unknown.

##### Etymology.

The name of this new species, stellatus, is derived from the star-shaped reticulations covering its dorsal shields.

##### Remarks.

The new species is similar to *A. apophysis* Chen & Jin, 2021 in having one pointed apophysis on palp basifemur. However, it can be distinguished from *A. apophysis* by the following characteristics: (1) dorsal shields covered with star-shaped reticulations, which are composed of papillae and reticulations (vs general reticulations in *A. apophysis*); (2) median shield large and with three pairs of simple setae (*c_1_*, *d_1_*, *e_1_*) (vs small and without in *A. apophysis*); (3) hysterosoma lateral plates absent (vs present in *A. apophysis*); (4) subcapitulum with reticulations (vs without in *A. apophysis*).

The new species is similar to *A. anastosi* Smiley, 1992 in having two apophysis on palp telofemur. Nevertheless, it differs from *A. anastosi* in the following characteristics: (1) dorsal shields covered with star-shaped reticulations, which are composed of papillae and reticulations (vs general reticulations in *A. anastosi*); (2) median shield with three pairs of simple setae (*c_1_*, *d_1_*, *e_1_*) (vs two (*c_1_*, *d_1_*) in *A. anastosi*); (3) hysterosoma lateral plates absent (vs present in *A. anastosi*); (4) palp basifemur with one pointed apophysis (vs without in *A. anastosi*).

##### Material examined.

***Holotype***: China • 1 ♀ (slide no. QH-CU-2020071901), Sanjiangyuan National Nature Reserve, Qinghai Province; 35°13'17"N, 107°57'10"E; 3027 m a.s.l.; 19 July 2020; collected from moss by Dong-Dong Li and Hai-Tao Li. ***Paratypes***: China • 1 ♀ (QH-CU-2019072001), Qingyanggou Section, Qilian Mountain Nature Reserve, Qinghai Province; 38°9'53"N, 100°26'10"E; 3162 m a.s.l.; 20 July 2019; collected from ground moss by Qian-Fen Zheng • 2 ♀ (XJ-CU-201907060601–XJ-CU-201907060602), Kanas National Nature Reserve, Buerjin County, Xinjiang Uygur Autonomous Region; 48°30'40"N, 87°10'16"E; 1330 m a.s.l.; 6 July. 2019; collected from moss by Jian-Xin Chen.

##### Distribution.

China (Qinghai Province and Xinjiang Uygur Autonomous Region).

### Key to species of *Armascirus* in China (adult females)

**Table:** 

1	Palp basifemur with one pointed apophysis	**2**
–	Palp basifemur without pointed apophysis	**3**
2	Proterosomal and median shields covered with star-shaped reticulations, median shield large and with three pairs of simple setae (*c_1_*, *d_1_* and *e_1_*), lateral plates absent	***A. stellatus* Chen & Jin, sp. nov**.
–	Proterosomal and median shields covered with general reticulations, median shield small and without setae, lateral plates present	***A. apophysis* Chen & Jin, 2021**
3	Palp telofemur with two apophyses, five pairs of genital setae (*g_1_*–*g_5_*)	***A. yulongensis* Chen & Jin, 2021**
–	Palp telofemur with one apophysis, four pairs of genital setae (*g_1_*–*g_4_*)	**4**
4	Hysterosomal lateral plates absent	***A. bison* (Berlese,1888)**
–	Hysterosomal lateral plates present	**5**
5	Lateral plates with longitudinal striae	***A. jini* Liu, Yi & Guo, 2015**
–	Lateral plates with reticulations	**6**
6	Median shield with setae (*d_1_*)	***A. taurus* (Kramer, 1881)**
–	Median shield without setae	***A. kuandianensis* Chen & Jin, sp. nov**.

## Supplementary Material

XML Treatment for Armascirus

XML Treatment for Armascirus kuandianensis

XML Treatment for Armascirus stellatus

## Additional information

### Use of AI

No use of AI was reported.

### Author ORCIDs

Jian-Xin Chen https://orcid.org/0000-0001-8787-6678

Mao-Yuan Yao https://orcid.org/0000-0002-5475-046X

Tian-Lei Liu https://orcid.org/0000-0002-1524-8020

You-Fang Wu https://orcid.org/0000-0001-7968-5345

Jian-Jun Guo https://orcid.org/0000-0002-0523-6704

Dao-Chao Jin https://orcid.org/0000-0003-2727-5621

### Data availability

All of the data that support the findings of this study are available in the main text.
